# Reduced Expression of CD27 by Collagenase Treatment: Implications for Interpreting B Cell Data in Tissues

**DOI:** 10.1371/journal.pone.0116667

**Published:** 2015-03-10

**Authors:** Chanjuan Shen, Huanbin Xu, Xavier Alvarez, Andrew A. Lackner, Ronald S. Veazey, Xiaolei Wang

**Affiliations:** Division of Comparative Pathology, Tulane National Primate Research Center, Covington, Louisiana, United States of America; Tulane University, UNITED STATES

## Abstract

Surface markers have been used to identify distinct cell subpopulations and to delineate various stages of maturation or activation of lymphocytes. In particular CD27 is used for delineation of naïve and memory B cell populations, and is readily detected by flow cytometry. We here used flow cytometry to examine the expression of CD27 on lymphocytes isolated from various tissues of rhesus macaques, and found its expression was consistently low to absent on intestinal cell suspensions. However, immunohistochemistry revealed abundant CD27+ cells in intestinal tissue sections. Further investigation showed the marked loss of CD27 expression on processed intestinal cells was due to collagenase digestion of intestinal tissues, yet CD27 expression was recoverable within hours of cell isolation. By combining confocal microscopy, we confirmed that only a fraction of B cells express CD27, in contrast to expression on all T cells from tissues examined including the gut. Taken together, our results suggest that CD27 may be a memory marker for B cells, but not for T cells, since essentially all CD3 T cells expressed CD27. In summary, it is important to consider the influence of isolation procedures on cell surface expression of phenotypic markers, especially when examining tissue-resident lymphocytes by flow cytometry.

## Introduction

There are large numbers of lymphocytes constitutively present in the intestinal mucosa, which have distinct phenotypic characteristics from those of peripheral lymphoid tissues. For example, regional or tissue-resident memory T cells (Trms) do not express CCR7, but have high CD103 expression that is regulated by TGF-β, and usually co-express CD69 [1,2]. In addition, CD27, a member of the tumor-necrosis-factor-receptor (TNFR) superfamily, has been shown to be critical for T cell expansion, survival, and induction of long-term memory [3,4], and also contributes to germinal center formation, B cell activation, and antibody production [5–7]. Therefore, measuring CD27 expression on intestinal both T and B lymphocytes may be important for assessing regional immune responses.

Non-human primates are widely used in AIDS research because they most closely resemble humans in their physiology and immunology. The ability to distinguish naïve and memory subsets in macaques led to the discovery that simian immunodeficiency virus (SIV) rapidly and selectively infects and eliminates “memory” CD4+ T cells, particularly in mucosal tissues [8–10], findings that were confirmed in HIV-infected patients [11,12]. Of note, among 30% of those HIV-exposed individuals that seem resistant to infection despite multiple, long-term exposures [13,14], the presence of high levels of HIV-neutralizing sIgA in the genital tract and HIV-reactive T cells in the cervix appear to correlate with resistance to infection [13–15]. Although tissue-resident memory T cells have been intensely studied, few studies have characterized the local resident lymphocytes B-cells, which may provide better understanding of generating mucosal humoral immune responses, and improving mucosal vaccination strategies to prevent HIV infection and/or disease progression. The CD27 antigen has been defined as a key marker for identifying memory B cells [16], and its signaling promotes the differentiation of memory B cells into plasma cells [17]. Therefore, examining CD27 expression levels is critical for monitoring B cell maturation and development in SIV/HIV infection and other diseases.

Multicolor flow cytometry is a powerful tool to exquisitely quantify even rare cell populations, and allows identification and characterization of novel cell subsets. However, examining cells from mucosal tissues, such as intestines or reproductive tissues requires digestion and processing into single cell suspensions, and certain digestion techniques may dramatically alter expression of surface markers through downregulation, upregulation, or cleavage of surface proteins. Here we used flow cytometry and microscopy to examine and compare CD27 expression on lymphocytes isolated from various tissues including the intestine in rhesus macaques, and evaluated the influence of cell isolation procedures on its expression.

## Materials and Methods

### Animals and Ethics Statement

The eight rhesus macaques (*Macaca mulatta*) used in this study were housed at the Tulane National Primate Research Center in accordance with the Association for Assessment and Accreditation of Laboratory Animal Care International standards. All animals were received standard primate feed as well as fresh fruit and enrichment daily, and had continual access to water. Animal welfare was monitored daily. All studies were reviewed and approved by the Tulane University Institutional Animal Care and Use Committee (IACUC) of Tulane University (protocol numbers P0049 and P0199). Animal housing and studies were carried out in strict accordance with the recommendations in the Guide for the Care and Use of Laboratory Animals of the National Institutes of Health (NIH, AAALAC #000594) and with the recommendations of the Weather all report; “The use of non-human primates in research”. All clinical procedures, including administration of anesthesia and analgesics, were carried out under the direction of a laboratory animal veterinarian. All procedures were performed under anesthesia using ketamine, and all efforts were made to minimize suffering and stress, improve housing conditions, and to provide enrichment opportunities (e.g., objects to manipulate in cage, varied food supplements, foraging and task-oriented feeding methods, interaction with caregivers and research staff). Our standard single cages are 4.3^2^ ft x 36”. However, animals are usually housed in pairs in larger cages, which at a minimum provide at least 4.3ft^2^ x 30” per animal. If animals are 10 kg or more, twice this amount of space is allocated per animal. The standard method of euthanasia for nonhuman primates was used in this study, which is complete anesthesia with Telazol and buprenorphine followed by a lethal intravenous dose of sodium pentobarbital (approximately 156 mg/kg) via the intracardiac or intravenous route (IACUC approved TNPRC SOP3.23). This method is consistent with the recommendation of the American Veterinary Medical Association Guidelines on Euthanasia. Tulane University complies with NIH policy on animal welfare, the Animal Welfare Act, and all other applicable federal, state and local laws.

### Tissue collection, cell isolation and flow cytometry

All tissues used in this study were collected from necropsy. EDTA-blood, portions of spleen, mesenteric lymph nodes, and 6 to 8-cm long sections of the jejunum were also collected fresh in complete RPMI 1640 medium containing 5% FCS, 100IU/mL penicillin/streptomycin, 2mM glutamine, and 25mM Hepes buffer (R5 medium) and immediately transported to the laboratory for lymphocyte isolation. Peripheral blood mononuclear cells (PBMC) were prepared by density gradient centrifugation with Lymphocyte Separation Media (MP Biomedicals, LLC, Santa Ana, CA) according to manufacturers instructions. Lymphocytes were isolated from spleen and lymph node by gently cutting and pressing tissues through nylon mesh screens as routinely performed [18,19]. Lymphocytes from the intestine were initially isolated using procedures previously described [20,21]. Briefly, intestinal pieces were subjected to two sequential incubations with 1 mM/mL EDTA (EMD Chemicals Inc., Germany) in HBSS with 5% FCS for 30 min at 37°C in an Environ-Shaker with 300 RPM to remove the epithelium. Remaining tissues were digested twice with 0.75 mg/mL collagenase type II (Sigma, St Louis, MO) in R5 medium for 30 min at 37°C in a shaker with 300 RPM to digest and extract lymphocytes from lamina propria. Samples were spun (400g, 10 min) and supernatents discarded after each digestion, and cell pellets from all incubations were pooled and resuspened in R5 medium, and gently layer onto a bilayer Percoll gradient containing 60% isotonic Percoll (bottom) and 35% Percoll (top), and centrifuged 20 min at 1000g. The interface containing the lymphocytes was removed, and resuspended in R5 medium at 1x10^7^/ml for flow cytometry staining.

To prove the collagenase digestion procedures affected cell surface marker expression, PBMCs, spleen and lymph node were subjected to identical serial incubations in EDTA and collagenase as above. We also examined the effects of treatment with EDTA or collagenase alone on PBMCs subjected to two serial incubations with either EDTA or collagenase as used in intestinal processing protocol. Further, parallel intestinal sections were similarly obtained with and without EDTA or collagenase treatment compared to cells obtained simply by finely mincing intestinal tissues to release intestinal cells without digestion (mechanical disruption). From one animal, parallel intestinal segments were isolated using mechanical, EDTA alone, and collagenase digestion alone and incubated with collagenase at different concentrations and durations of exposure.

To assess recovery of CD27 after collageanse digestion, aliquots of intestinal cell suspensions were stained for flow cytometry immediately after preparation, and remaining cells were incubated in complete RPMI media containing 10% FBS, 1% Pen/Strep, 1% Hepes, 1% glutamine and 1% β-ME in a 37°C in a CO_2_ incubator. From these, aliquots were serially harvested for staining and analysis at 4, 8, 12, and 24 hr by flow cytometry.

For routine staining, blood and spleen samples were stained using a whole blood lysis protocol as previously described [19,22]. Prepared intestinal cell suspensions were always adjusted to 10^7^ cells / mL and 100 μL aliquots (10^6^ cells) were stained with appropriately diluted concentrations of antibodies at 4°C for 30 min. Stained cells were then washed in PBS and fixed with BD Stabilizing Fixative (BD Biosciences). At least 10,000 lymphocytes for each sample were acquired on BD LSRFortessa (Becton Dickinson). Data were analyzed with Flowjo software (Tree star, Inc.). Fluorochrome-conjugated monoclonal antibodies used for flow cytometry included CD27-PE-Cy7 (Clone M-T271, BD Biosciences) or CD27-PE-Cy7 (clone O323, eBiosciences), CD20-APC-H7 (2H7, BD Biosciences) and CD3-PE (SP34–2, BD Biosciences), and LIVE/DEAD FIXABLE AQUA DEAD CE (Invitrogen).

### Immunohistochemistry staining

Standard immunohistochemistry (IHC) protocols were used for detection of CD27, CD20 (Clone L26, Dako Inc), and CD3 (rabbit anti-human, Dako Inc.) on frozen sections of lymph node and intestinal tissues as previously described [19]. Two purified monoclonal mouse anti-human CD27 antibodies were tested here; one from BD Biosciences (clone M-T271), and another from eBiosciences (clone O323), both of which have been reported to cross-react with rhesus macaques [22–24]. Briefly, tissues collected from necropsy were embedded in optimum cutting temperature compound (Tissue-Tek O.C.T. Compound, Sakura Finetek), snap frozen in dry-ice cooled 2-methylbutane and stored at -80°C until sectioning. Adjacent 5 mm sections were cut using a Microm HM560 Cryostat (Thermo Scientific Inc.), adhered to glass slides, fixed with cold acetone for 10 min, then washed twice in phosphate buffered saline (PBS) for IHCs. Slides were then blocked with peroxidase blocking reagent (Dako Inc.) for 10 min at room temperature (RT), washed in PBS, blocked with serum-free protein block (Dako Inc.) for 30 min, then incubated with the appropriately diluted primary antibodies for 1 hour. Slides were then washed with PBS, processed with a Vectastain ABC peroxidase kit (Vector Laboratories, Inc) and developed with 3,3-diaminobenzidine DAB (Biocare Medical). Additional cryostat sections from three macaques were digested with 0.75 mg/mL collagenase for 10 min at 37°C before IHC staining. Other sections were identically stained with isotype serum controls to exclude false positive signals. All images were acquired by a Leica DMLB microscope.

### Tri-color confocal microscopy

Three-color immunofluorescence staining for CD27, CD3 and CD20 was performed on cryostat sections as described previously [19]. Briefly, tissue sections were fixed with cold acetone, blocked with serum-free protein block (Dako Inc.), and incubated with appropriately diluted primary antibodies, as above, then then incubated with Alexa Fluor 488 (green) labeled goat anti-mouse IgG1 for CD27, Alexa Fluor 568 (red) labeled goat anti-mouse IgG2a for CD20, and Alexa Fluor 647 (blue) labeled anti-rabbit IgG (H+L) for CD3 respectively. Sections were then stained with secondary antibodies to detect CD27 (green), CD20 (red) and CD3 (blue) (Invitrogen, Carlsbad, CA). Slides were then coverslipped with fluorescent mounting medium (Dako, Inc.). Confocal microscopy was performed using a Leica TCS SP2 confocal microscope equipped with three lasers (Leica Micro-systems, Exton, PA). Individual optical slices were collected at 512×512 pixel resolution. Adobe Photoshop (version CS6) was used to assign colors to the channels collected.

### Statistics

Graphical presentation and statistical analysis of the data were performed using GraphPad Prism 6.0 (GraphPad Software Inc., SanDiego, CA). Comparisons between groups were analyzed by a two-tailed paired t-test. P values ≤ 0.05 were considered statistically significant. All experiments were performed in three different animals unless otherwise stated.

## Results

### Collagenase treatment significantly reduces CD27 expression on lymphocytes

**Fig 1:**
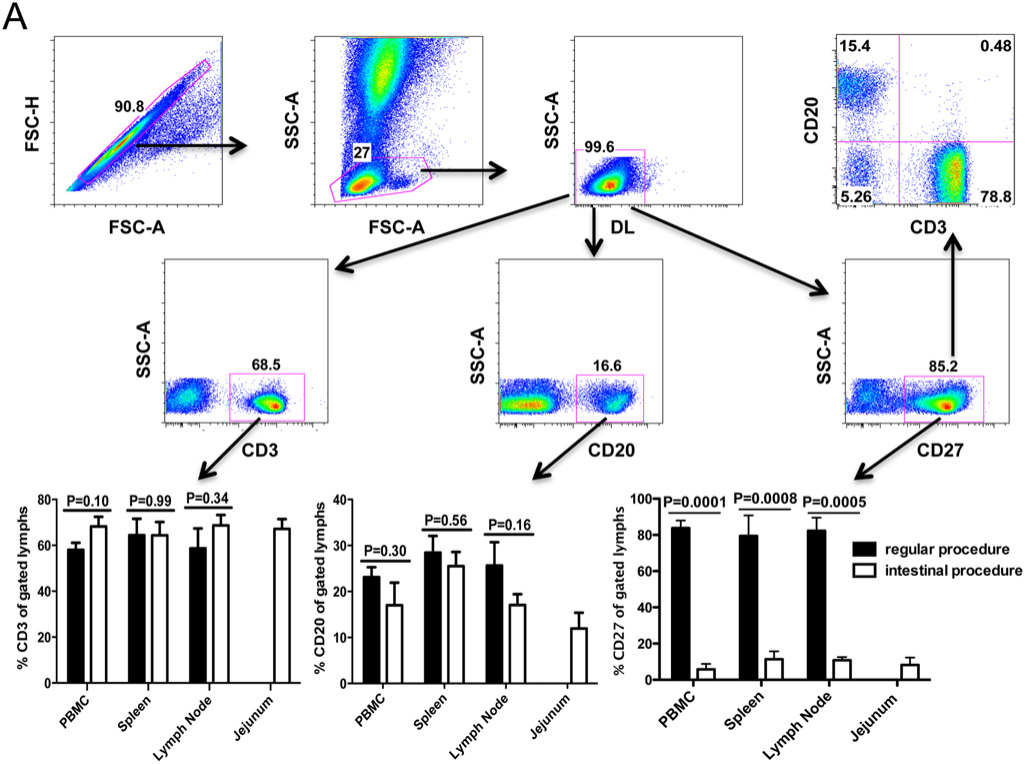
Expression of CD27 on lymphocytes isolated from various tissues of rhesus macaques detected by flow cytometry. (A) Representative plots generated from whole blood samples showing gating strategy for analysis of CD27+ lymphocytes isolated from all tissues. Specifically, singlets were gated first followed by lymphocytes, and then live lymphocytes (DLneg) were selected for further analysis of CD3+, CD20+, CD27+ as shown as in A; (B) Comparison of percentages of various cells including CD3+, CD20+ and CD27+ after different processing of the same tissues and gating through live lymphocytes. Note that significantly decreased CD27 expression is only detected on lymphocytes subjected to collagenase digestion procedures when compared to no digestion. In contrast, there are no changes in expression of CD3 or CD20 on lymphocytes indicating this is selective for CD27. Bars represent mean percentages ± SEM in each group (n = 7), P values ≤ 0.05 were considered statistically significant.

We first examined the expression of CD27 on the PBMCs and the lymphocytes processed from spleen and lymph nodes immediately *ex vivo* (without processing), and found most lymphocytes expressed CD27 in all tissues examined. Among CD27+ cells, most were T cells (CD3+), and fewer were B cells (CD20+), and a very small population of non-T /non-B lymphocytes expressed CD27 (Fig. 1A). Surprisingly, significantly fewer CD27+ lymphocytes were found in the same tissues after processing with the collagenase type II digestion. As shown as in Fig. 1B, average percentages of CD27+ lymphocytes markedly decreased from 83.8% to 5.8% in PBMCs, from 79.5% to 11.4% in spleen, and from 82.3% to 10.8% in lymph nodes. For comparison, an average of 8.2% of intestinal cells co-expressed CD27. In contrast, the digestion procedures had no effect on CD3 and minimal effects on CD20 expression, as indicated by no significant differences in percentages before and after treatment (Fig. 1B).

**Fig 2:**
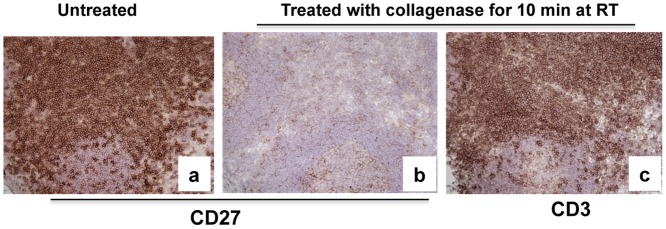
Comparison of CD27 expression in lymph node tissue sections before and after collagenase treatment. Note marked loss of CD27 signal is detected following collagenase treatment of adjacent frozen sections of the same lymph node (a and b). In contrast, collagenase treatment has no effect on CD3 expression (c).

To determine which treatment step resulted in loss of CD27 expression we also treated PBMCs either subjected to two serial incubations with EDTA alone or collagenase twice for 30 min at 37°C in a shaker with 300 RPM as used in intestinal processing protocol. The results showed that only the collagenase treatment resulted in loss of CD27 expression on PBMC (data not shown). We further confirmed this result directly by immunohistochemistry (IHC) on frozen sections to detect and compare levels of CD27 on collagenase treated and untreated frozen tissue sections. As shown in Fig. 2, collagenase-treated tissue sections had few positive cells with minimal CD27 signal (Fig. 2b). In contrast, sections not pre-treated with collagenase had abundant CD27 expression (Fig. 2a). Pre-treatment of sections with collagenase had no effect on CD3 expression (Fig. 2c). These findings confirmed the flow cytometry results showing that only CD27, but not CD3 were reduced by collagenase treatment. Combined, these results clearly showed collagenase treatment could essentially abolish CD27 expression on lymphocytes.

### Rapid recovery of CD27 expression on collagenase treated lymphocytes

**Fig 3:**
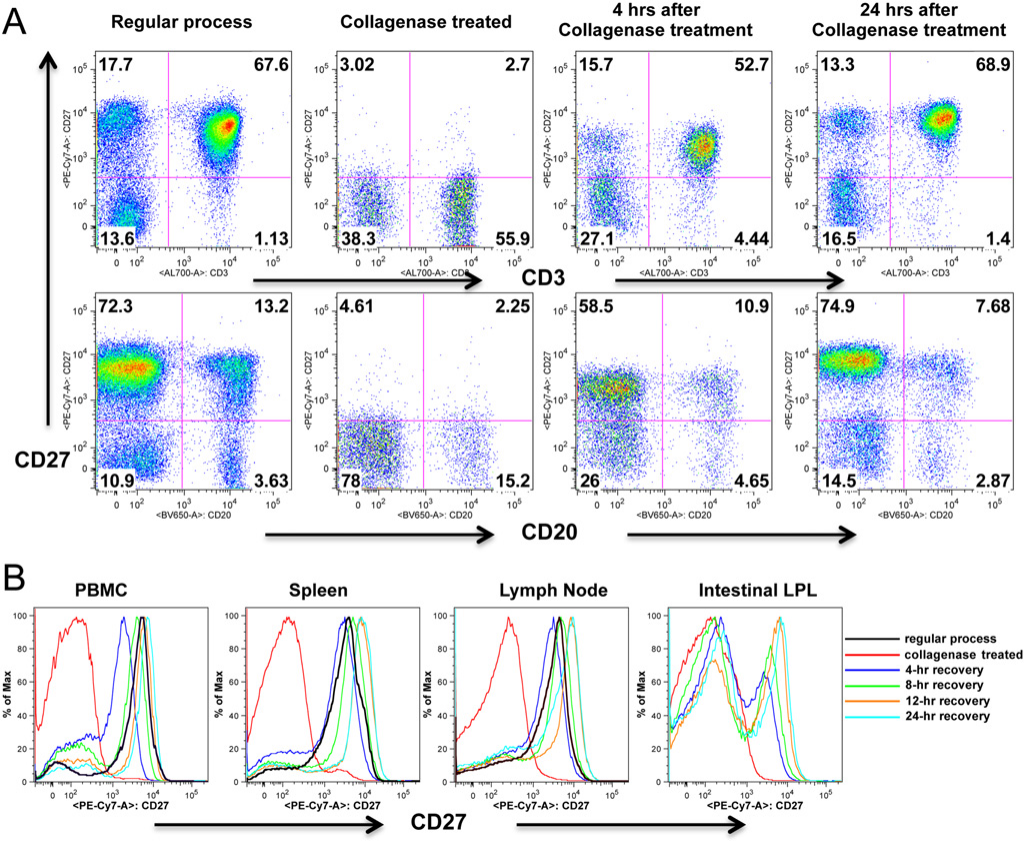
CD27 expression recovers on lymphocytes following treatment with collagenase. (A) Dot plots show collagenase treated PBMCs lose surface CD27 expression, but expression is almost entirely recovered after 4, and entirely recovered after 24 hrs rest / incubation. Numbers in each quadrant indicate the percentage of total live lymphocytes as indicated in Fig. 1. (B) Rapid recovery of CD27 expression on collagenase treated cells occurs in all tissues including intestinal tissues. Histograms are representative of four independent experiments.

To determine if CD27 expression would recover after collagenase treatment, collagenase treated lymphocytes were incubated with C10 medium at 37°C in CO2 incubator for 4, 8, 12, 16 and 24 hrs, and found a rapid recovery of CD27 expression. As shown as in Fig. 3, after 4 hours incubation, CD27 expression on collagenase treated lymphocytes were comparable to untreated lymphocytes. In fact, CD27 expression was apparently completely recovered by 8 hrs after treatment, and was maintained at pre-treatment levels through 24 hrs of incubation in all tissues examined.

### Identification and localization of CD27+ cells in the gut

Data from the above experiments suggested the low level of CD27 expression detected on intestinal lymphocytes was an artifact of collagenase treatment involved in preparation of single cell suspensions.

**Fig 4:**
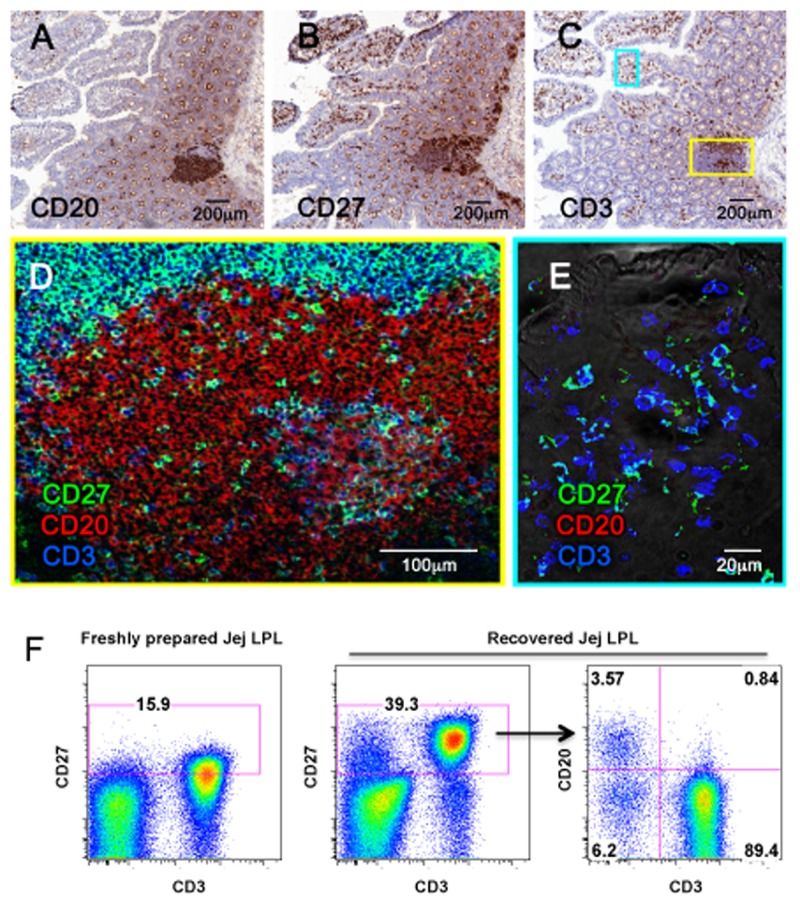
Identification and localization of CD27+ cell subsets in intestinal tissues. Immunohistochemistry staining intestinal B cells (CD20+) (A), CD27+ cells (B), and T cells (CD3+) (C) on the serial adjacent sections. Three-color confocal microscopy images show that CD27+ B cells are mainly located in lymphoid aggregates in the deeper lamina propria of the gut (D), whereas CD27+ cells in the intestinal villi are all T cells (E). Flow cytometry of intestinal cell suspensions confirms most CD27+ cells are CD3+ T cells, with fewer percentages of CD20+ B cells co-expressing CD27 (F).

Immunohistochemistry staining showed that CD20+/B cells were mostly found in gut-associated lymphoid tissues (GALT) in the lamina propria (Fig. 4A), whereas CD27+ cells and CD3+/T cells were evenly spread throughout the lamina propria of the intestinal villi and in the GALT (Fig. 4B and C). Multicolor microscopy further demonstrated most CD27+ cells in the villi were T cells (CD3+) (Fig. 4E), whereas CD27+ B cells (CD20+) were only found in GALT (Fig. 4D). Flow cytometry data from incubated and CD27-recovered intestinal lymphocytes also suggested most intestinal CD27+ lymphocytes were T cells, as fewer B cells and rare non-T, non-B cells expressed CD27 as shown as in Fig. 4F.

## Discussion

Immunologic memory to previously encountered antigens is a hallmark of the adaptive immune system, which confers long-term protection, and is the basis for efficacious vaccines. Therefore, distinguishing naïve and memory cell responses has greatly facilitated the investigation of lymphocyte function and immunologic responses. The antigen CD27 is of particular interest for immunology and vaccine research, as it is well documented as a general marker for memory B cells in the blood of humans [17], and it is required for generation and long-term maintenance of T cell immunity [3]. Recent studies have also confirmed naïve and memory B cell populations of macaques can be differentiated by surface level expression of CD27 [23]. However, here we show it is crucial to consider the effects of cell isolation procedures when examining tissue-derived cells.

In this study, we used multicolor flow cytometry to compare the expression of CD27 on lymphocytes isolated from peripheral lymphoid tissues including lymph nodes and spleen from rhesus macaques, and found over 80% of lymphocytes expressed CD27 in these tissues (Fig. 1). However, and in contrast to CD3+ T cells, which all expressed CD27 in these tissues, only a fraction of peripheral B cells expressed CD27 (Fig. 3A). Three-color confocal microscopy further confirmed these results in lymph nodes, and also demonstrated CD27+ B cells were mainly restricted to germinal centers (See S1 Fig. for more detailed information). However, flow cytometry data on isolated lymphocytes from intestinal tissues revealed very low CD27 expression, and practically no CD27 expression was found on intestinal B cells, consistent with previous reports [24,25]. Surprisingly however, immunohistochemistry on frozen sections revealed abundant CD27+ cells in the both lamina propria and GALT (Fig. 4B), whereas very few cells were detected in collagenase II digested single cell suspensions of gut tissues by flow cytometry. Further, we compared both clones (M-T271 and O323) of anti-human CD27 in macaque tissues and found that both cross react, and we detected no significant difference in levels of expression with either clone (data not shown). The results presented here for both flow cytometry and immunochemistry were generated using clone M-T271.

Although we and others have previously shown that most collagenase-based intestinal digestion procedures do not result in changes of expression of several cell surface markers [19,20,26], apparently this is not the case for CD27, as evidenced by the disparate results of the flow cytometry and immunohistochemistry for CD27 on intestinal sections as shown as Fig. 4. To confirm this, sequential 1 mg or so segments of jejunum from one animal were subjected to various isolation and digestion procedures to determine the effects on cell yield and CD27 expression. We found even a single 30 min exposure, or low concentrations (0.1mg/ml) of collagenase resulted in marked loss of CD27 expression on intestinal lymphocytes (See S2 Fig. for more detailed information). Nonetheless, collagenase treatment dramatically improves lymphocyte release from intestinal tissues, especially for B cells evidenced by samples 1 and 2, and increasing collagenase concentrations result in increased total cell recovery, yet complete loss of CD27 staining. Thus, intestinal tissues clearly have abundant CD27+ lymphocytes, yet this is almost completely masked by tissue digestion techniques, even when using brief collagenase exposures or concentrations. Further, processing PBMCs and lymphocytes from blood, lymph node, and spleen using identical procedures as for intestinal cell isolation clearly showed significant decreases in surface expression of CD27, despite complete preservation of the other makers in the current study (Fig. 1). Finally, digesting tissue sections with collagenase alone definitively showed that collagenase was responsible for the loss of CD27 expression in cells and tissues, whereas collagenase had no effect on CD3+ expression (Fig. 2). Combined, our results clearly showed collagenase II selectively abolishes CD27 expression on the surface of lymphocytes of primates. A recent study in mice confirms these results, as Chen et al demonstrated that both collagenase type I and IV have similar selective effects on CD27 expression in murine cells [27].

Although collagenase-treated lymphocytes lost most of their surface CD27 expression, its expression can be fully recovered within hours of rest/incubation of cells. As shown as in Fig. 3, levels of CD27 expression on collagenase-treated lymphocytes PBMC and peripheral lymph node cells were comparable to untreated lymphocytes within 4 hours of incubation. Flow cytometry also showed recovery of CD27 expression on most intestinal T cells and some B cells as shown as in Fig. 4F, similar to our observations of CD27 detection by immunochemistry in the gut (Fig. 4A-E). Notably, in the intestine, substantial percentages of B cells expressed CD27, but these cells were restricted to germinal centers, similar to the expression patterns observed in peripheral lymph nodes (Fig. 4). This supports the hypothesis that CD27 signaling in B cells play a direct role in B cell memory commitment and differentiation, and contributes to germinal center formation [5,6,28]. However CD27+ cells in the intestinal lamina propria were almost exclusively CD3+ T cells, suggesting this molecule may play a different role in T cells. In summary, these results clearly demonstrate that there are abundant CD27+ lymphocytes in the gut, yet caution must be applied when interpreting flow cytometry data on collagenase-digested cell suspensions from intestine or other tissues, as CD27 expression is markedly, and selectively reduced on lymphocytes after collagenase treatment.

## Supporting Information

S1 Fig Identification of CD27+ cells in the stich image of a whole lymph node of rhesus macaques. The images are taken by confocal microscopy. Note that nearly all T cells (CD3+ in blue) co-expressed CD27 (in green), and a small fraction of B cells (CD20+ in red) co-expressed CD27 particularly residing in the germinal center of each follicle area.
(TIF)

S2 Fig Effects of varying the dose and duration of collagenase treatment on intestinal cell yield and surface antigen expression in one rhesus macaque. Parallel segments of jejunum from the same animal were exposed to mechanical digestion, EDTA or collagenase alone, or to varying concentrations and durations of collagenase treatment. The numbers above each set of dot plots correspond to the number and condition in table below. Note that CD27 expression is markedly decreased even when low or short durations to collagenase are used. However, collagenase digestion is essential to isolate lymphocytes as evidenced by increasing proportions of B and T cells compared to EDTA or mechanical process alone. Numbers in each quadrant indicate the percentage of total live lymphocytes as indicated in Fig. 1.
(TIF)

## References

[1] Shin H, Iwasaki A (2013) Tissue-resident memory T cells. Immunol Rev 255: 165–181. 10.1111/imr.12087 23947354 PMC3748618

[2] Cauley LS, Lefrancois L (2013) Guarding the perimeter: protection of the mucosa by tissue-resident memory T cells. Mucosal Immunol 6: 14–23. 10.1038/mi.2012.96 23131785 PMC4034055

[3] Hendriks J, Gravestein LA, Tesselaar K, van Lier RA, Schumacher TN, et al (2000) CD27 is required for generation and long-term maintenance of T cell immunity. Nat Immunol 1: 433–440. 11062504 10.1038/80877

[4] Hintzen RQ, Lens SM, Lammers K, Kuiper H, Beckmann MP, et al (1995) Engagement of CD27 with its ligand CD70 provides a second signal for T cell activation. J Immunol 154: 2612–2623. 7876536

[5] Nagumo H, Agematsu K, Shinozaki K, Hokibara S, Ito S, et al (1998) CD27/CD70 interaction augments IgE secretion by promoting the differentiation of memory B cells into plasma cells. J Immunol 161: 6496–6502. 9862673

[6] Raman VS, Akondy RS, Rath S, Bal V, George A (2003) Ligation of CD27 on B cells in vivo during primary immunization enhances commitment to memory B cell responses. J Immunol 171: 5876–5881. 14634097 10.4049/jimmunol.171.11.5876

[7] Arens R, Tesselaar K, Baars PA, van Schijndel GM, Hendriks J, et al (2001) Constitutive CD27/CD70 interaction induces expansion of effector-type T cells and results in IFNgamma-mediated B cell depletion. Immunity 15: 801–812. 11728341 10.1016/s1074-7613(01)00236-9

[8] Li Q, Duan L, Estes JD, Ma ZM, Rourke T, et al (2005) Peak SIV replication in resting memory CD4+ T cells depletes gut lamina propria CD4+ T cells. Nature 434: 1148–1152. 15793562 10.1038/nature03513

[9] Mattapallil JJ, Douek DC, Hill B, Nishimura Y, Martin M, et al (2005) Massive infection and loss of memory CD4+ T cells in multiple tissues during acute SIV infection. Nature 434: 1093–1097. 15793563 10.1038/nature03501

[10] Veazey RS, Mansfield KG, Tham IC, Carville AC, Shvetz DE, et al (2000) Dynamics of CCR5 expression by CD4(+) T cells in lymphoid tissues during simian immunodeficiency virus infection. J Virol 74: 11001–11007. 11069995 10.1128/jvi.74.23.11001-11007.2000 PMC113180

[11] Mehandru S, Poles MA, Tenner-Racz K, Horowitz A, Hurley A, et al (2004) Primary HIV-1 infection is associated with preferential depletion of CD4+ T lymphocytes from effector sites in the gastrointestinal tract. J Exp Med 200: 761–770. 15365095 10.1084/jem.20041196 PMC2211967

[12] Brenchley JM, Schacker TW, Ruff LE, Price DA, Taylor JH, et al (2004) CD4+ T cell depletion during all stages of HIV disease occurs predominantly in the gastrointestinal tract. J Exp Med 200: 749–759. 15365096 10.1084/jem.20040874 PMC2211962

[13] Kaul R, Trabattoni D, Bwayo JJ, Arienti D, Zagliani A, et al (1999) HIV-1-specific mucosal IgA in a cohort of HIV-1-resistant Kenyan sex workers. Aids 13: 23–29. 10207541 10.1097/00002030-199901140-00004

[14] Mazzoli S, Lopalco L, Salvi A, Trabattoni D, Lo Caputo S, et al (1999) Human immunodeficiency virus (HIV)-specific IgA and HIV neutralizing activity in the serum of exposed seronegative partners of HIV-seropositive persons. J Infect Dis 180: 871–875. 10438383 10.1086/314934

[15] Kaul R, Plummer FA, Kimani J, Dong T, Kiama P, et al (2000) HIV-1-specific mucosal CD8+ lymphocyte responses in the cervix of HIV-1-resistant prostitutes in Nairobi. J Immunol 164: 1602–1611. 10640781 10.4049/jimmunol.164.3.1602

[16] Klein U, Rajewsky K, Kuppers R (1998) Human immunoglobulin (Ig)M+IgD+ peripheral blood B cells expressing the CD27 cell surface antigen carry somatically mutated variable region genes: CD27 as a general marker for somatically mutated (memory) B cells. J Exp Med 188: 1679–1689. 9802980 10.1084/jem.188.9.1679 PMC2212515

[17] Agematsu K (2000) Memory B cells and CD27. Histol Histopathol 15: 573–576. 10809378 10.14670/HH-15.573

[18] Xu H, Wang X, Lackner AA, Veazey RS (2014) PD-1(HIGH) Follicular CD4 T Helper Cell Subsets Residing in Lymph Node Germinal Centers Correlate with B Cell Maturation and IgG Production in Rhesus Macaques. Front Immunol 5: 85 10.3389/fimmu.2014.00085 24678309 PMC3958750

[19] Wang X, Xu H, Alvarez X, Pahar B, Moroney-Rasmussen T, et al (2011) Distinct expression patterns of CD69 in mucosal and systemic lymphoid tissues in primary SIV infection of rhesus macaques. PLoS One 6: e27207 10.1371/journal.pone.0027207 22096538 PMC3212564

[20] Veazey RS, Rosenzweig M, Shvetz DE, Pauley DR, DeMaria M, et al (1997) Characterization of gut-associated lymphoid tissue (GALT) of normal rhesus macaques. Clin Immunol Immunopathol 82: 230–242. 9073546 10.1006/clin.1996.4318

[21] Veazey RS, Tham IC, Mansfield KG, DeMaria M, Forand AE, et al (2000) Identifying the target cell in primary simian immunodeficiency virus (SIV) infection: highly activated memory CD4(+) T cells are rapidly eliminated in early SIV infection in vivo. J Virol 74: 57–64. 10590091 10.1128/jvi.74.1.57-64.2000 PMC111513

[22] Das A, Xu H, Wang X, Yau CL, Veazey RS, et al (2011) Double-positive CD21+CD27+ B cells are highly proliferating memory cells and their distribution differs in mucosal and peripheral tissues. PLoS One 6: e16524 10.1371/journal.pone.0016524 21304587 PMC3029363

[23] Kuhrt D, Faith S, Hattemer A, Leone A, Sodora D, et al (2011) Naive and memory B cells in the rhesus macaque can be differentiated by surface expression of CD27 and have differential responses to CD40 ligation. J Immunol Methods 363: 166–176. 10.1016/j.jim.2010.09.017 20875419 PMC3357916

[24] Thomas MA, Demberg T, Vargas-Inchaustegui DA, Xiao P, Tuero I, et al (2014) Rhesus macaque rectal and duodenal tissues exhibit B-cell sub-populations distinct from peripheral blood that continuously secrete antigen-specific IgA in short-term explant cultures. Vaccine 32: 872–880. 10.1016/j.vaccine.2013.12.014 24374153 PMC3916903

[25] Titanji K, Velu V, Chennareddi L, Vijay-Kumar M, Gewirtz AT, et al (2010) Acute depletion of activated memory B cells involves the PD-1 pathway in rapidly progressing SIV-infected macaques. J Clin Invest 120: 3878–3890. 10.1172/JCI43271 20972331 PMC2964982

[26] Abuzakouk M, Feighery C, O'Farrelly C (1996) Collagenase and Dispase enzymes disrupt lymphocyte surface molecules. J Immunol Methods 194: 211–216. 8765174 10.1016/0022-1759(96)00038-5

[27] Chen Z, Chen X, Xu Y, Xiong P, Fang M, et al. (2014) Collagenase digestion down-regulates the density of CD27 on lymphocytes. J Immunol Methods. 10.1016/j.jim.2014.06.017 25066632

[28] Xiao Y, Hendriks J, Langerak P, Jacobs H, Borst J (2004) CD27 is acquired by primed B cells at the centroblast stage and promotes germinal center formation. J Immunol 172: 7432–7441. 15187121 10.4049/jimmunol.172.12.7432

