# Supplementary figures and images for: Reduced Expression of CD27 by Collagenase Treatment: Implications for Interpreting B Cell Data in Tissues
Source: PLoS One. 2015 Mar 10;10(3):e0116667. doi: 10.1371/journal.pone.0116667 (PMC4355594; doi:10.1371/journal.pone.0116667)

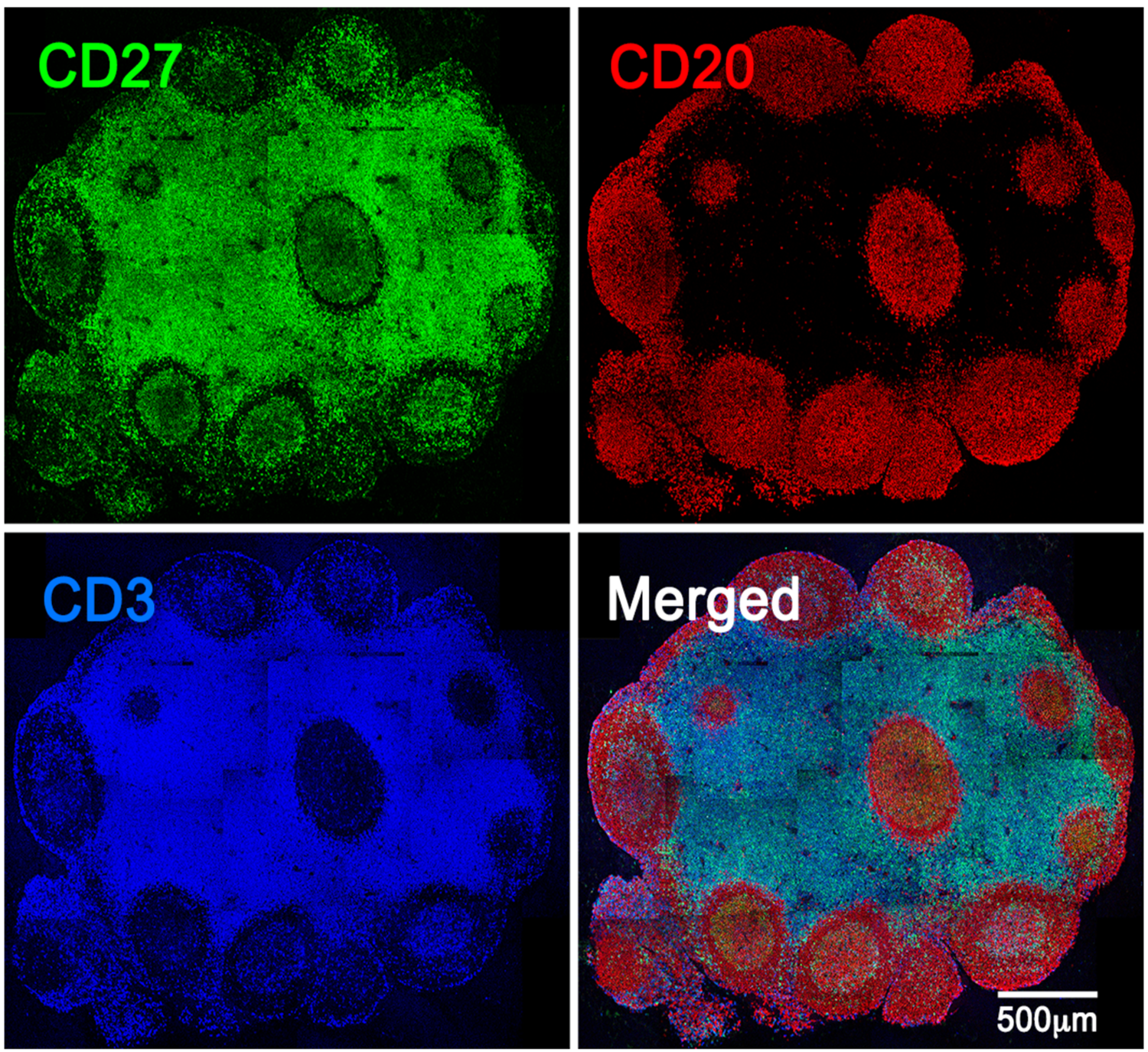

Supplement: S1 Fig — The images are taken by confocal microscopy. Note that nearly all T cells (CD3+ in blue) co-expressed CD27 (in green), and a small fraction of B cells (CD20+ in red) co-expressed CD27 particularly residing in the germinal center of each follicle area. (TIF) [file pone.0116667.s001.tif]

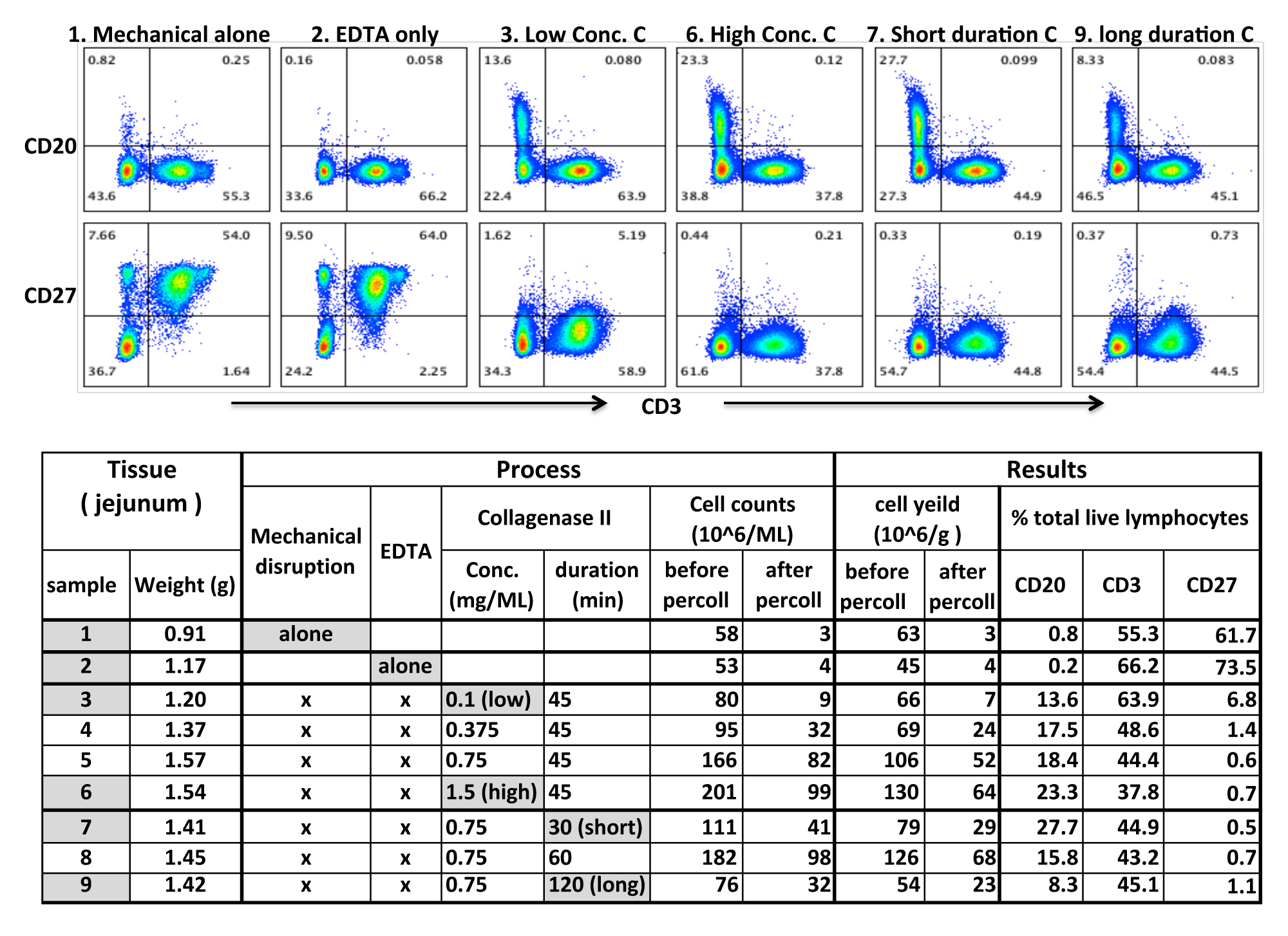

Supplement: S2 Fig — Parallel segments of jejunum from the same animal were exposed to mechanical digestion, EDTA or collagenase alone, or to varying concentrations and durations of collagenase treatment. The numbers above each set of dot plots correspond to the number and condition in table below. Note that CD27 expression is markedly decreased even when low or short durations to collagenase are used. However, collagenase digestion is essential to isolate lymphocytes as evidenced by increasing proportions of B and T cells compared to EDTA or mechanical process alone. Numbers in each quadrant indicate the percentage of total live lymphocytes as indicated in Fig. 1. (TIF) [file pone.0116667.s002.tif]
